# Lung Regulatory T Cells Express Adiponectin Receptor 1: Modulation by Obesity and Airway Allergic Inflammation

**DOI:** 10.3390/ijms21238990

**Published:** 2020-11-26

**Authors:** Patricia Ramos-Ramírez, Carina Malmhäll, Kristina Johansson, Mikael Adner, Jan Lötvall, Apostolos Bossios

**Affiliations:** 1 Krefting Research Centre, Department for Internal Medicine and Clinical Nutrition, Institute of Medicine, Sahlgrenska Academy, University of Gothenburg, SE-405 30 Gothenburg, Sweden; patricia.ramosramirez@liu.edu (P.R.-R.); carina.malmhall@gu.se (C.M.); kristina.johansson@ucsf.edu (K.J.); jan.lotvall@gu.se (J.L.); 2 Institute of Environmental Medicine, Karolinska Institutet, SE-171 65 Stockholm, Sweden; mikael.adner@ki.se; 3 Department of Respiratory Medicine and Allergy, Karolinska University Hospital, Huddinge, SE-141 86 Stockholm, Sweden; 4 Department of Medicine, Huddinge, Karolinska Institutet, SE-141 86 Stockholm, Sweden

**Keywords:** regulatory T cells, obesity, allergic inflammation, AdipoR1, Helios, adipose tissue, lung, obesity-related asthma, DIO-mouse model

## Abstract

Regulatory T cells (Tregs) decrease in the adipose tissue upon weight gain, contributing to persistent low-grade inflammation in obesity. We previously showed that adipose tissue Tregs express the adiponectin receptor 1 (AdipoR1); however, the expression in lung Tregs is still unknown. Here, we aimed to determine whether Helios^+^ and Helios^−^ Treg subsets expressed AdipoR1 in the lungs of obese mice and whether different obesity grades affected the expression upon allergic lung inflammation. For diet-induced obesity (DIO), mice were fed a high-fat diet (HFD) for up to 15 weeks (overweight), 21 weeks (obesity), and 26 weeks (morbid obesity). Overweight and morbidly obese mice were sensitized and challenged with ovalbumin (OVA) to induce allergic lung inflammation. The AdipoR1 expression was reduced significantly in the lung Helios^+^ and Helios^−^ Tregs of obese mice compared with lean mice. Airway allergic inflammation showed reduced AdipoR1 expression in lung Foxp3^+^ Tregs. Obesity significantly exacerbated the eosinophilic airway inflammation and reduced the number of Helios^+^ Tregs in lung and adipose tissue in the obesity-associated asthma model. Upon further weight gain, AdipoR1-expressing Tregs in the lungs of allergic mice were increased, whereas AdipoR1-expressing Tregs in adipose tissue were reduced. These data suggest that obesity-associated adipose tissue inflammation may exacerbate allergic inflammation by downregulating the AdipoR1^+^ Tregs in the lungs.

## 1. Introduction

Obesity is recognized by the World Health Organization (WHO) as an epidemic, as worldwide obesity has nearly tripled since 1975, reaching 650 million adults in 2016 [1]. Most of the body’s physiological functions are negatively affected by obesity, and obesity increases the risk of developing multiple diseases resulting in a public health threat [2]. Obesity is a frequently reported comorbidity in asthma, where obese asthmatics experience worse symptoms than lean asthmatics, including more severe and frequent exacerbations and a reduced response to asthma medications, which results in a significantly reduced quality of life [3]. A link between obesity and asthma was attributed to the excessive accumulation of adipose tissue, which may promote systemic inflammation that reaches distant sites, such as the lungs [4].

Adipose tissue, mainly white adipose tissue (WAT), is considered as an active endocrine and immune organ, with a diverse cellular composition capable of secreting a broad range of pro-inflammatory and anti-inflammatory adipokines, such as leptin and adiponectin, as well as cytokines that together trigger inflammation [4,5]. In addition, adipose tissue is infiltrated by various immune cells that also contribute to low-grade inflammation, generating a systemic effect [6]. It was recently demonstrated that adipose tissue is present within the airway wall and is related to BMI and the number of inflammatory cells in obstructive airway diseases [7].

Among adipose-related immune cells, Foxp3^+^ CD4^+^ regulatory T cells (Tregs) are of substantial interest as they play a critical role in metabolic homeostasis, regulating the inflammatory status of adipose tissue [8]. Adipose tissue in lean mice contains a large proportion of Foxp3^+^ Tregs (40%–80%) compared to lymphoid and non-lymphoid tissues (5%–15%), whereas the adipose tissue Tregs in obese mice are markedly reduced. Similarly, FOXP3 transcripts were reduced in human obese and morbidly obese omental fat compared with subcutaneous adipose tissue [9].

We recently showed that adipose tissue Tregs in lean mice expressed the adiponectin receptor 1 (AdipoR1). This expression was significantly reduced in a diet-induced obesity (DIO) mouse model [10], which linked two major anti-inflammatory mechanisms: adiponectin and Tregs. Low levels adiponectin were associated with asthma in adults [11]. Tregs are critical in limiting immune activation and inflammation in allergic diseases and asthma [12]; however, it is unknown if subsets of Tregs in the lung express AdipoR1 and if the expression is altered by obesity and pulmonary allergic inflammation.

Here, we investigated whether AdipoR1-expressing Tregs exist in the lungs of lean mice and whether weight gain and pulmonary allergic inflammation alter the expression in mouse models of DIO and airway allergic asthmatic inflammation.

## 2. Results

### 2.1. Obesity Reduces Airway Eosinophils

**Figure 1:**
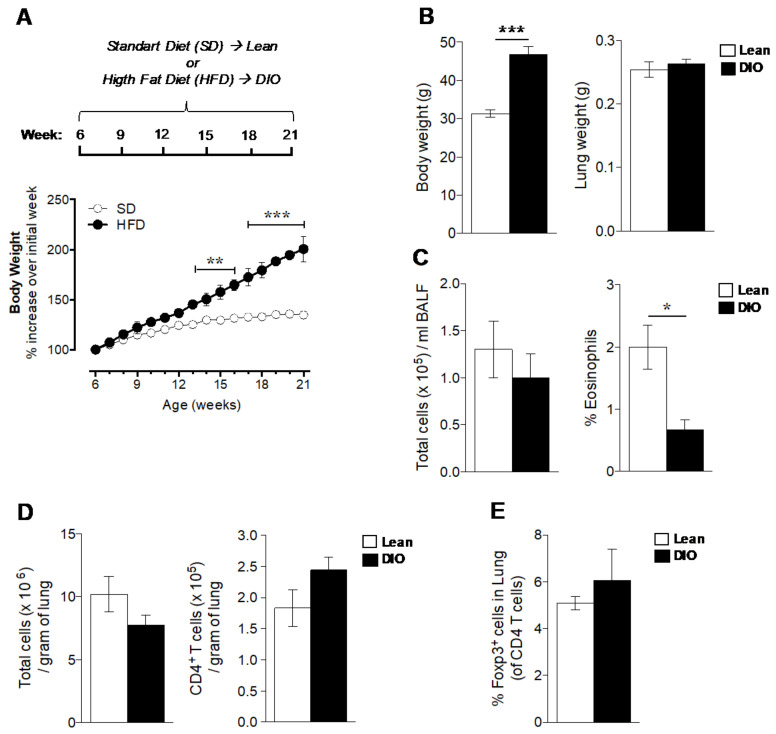
The effect of diet-induced obesity (DIO) on lung inflammation and the expression of Foxp3^+^ Tregs. (**A**) Experimental design for the DIO mouse model (upper) where mice on a high-fat diet (HFD) up to 21 weeks of age developed obesity compared to age-matched mice on a standard diet (bottom). (**B**) Body and lung weight in obese and lean mice at 21 weeks of age. (**C**) Total number of inflammatory cells and eosinophils in bronchoalveolar lavage fluid (BALF). (**D**) Total number of inflammatory cells and CD4^+^ T cells per gram of lung tissue. (**E**) Frequency of Foxp3^+^ cells within CD4^+^ T cells in the lungs. Tregs determined as CD3^+^ CD4^+^ Foxp3^+^ cells. Data are means ± SEM of n = 3–6 mice per group. Two-way ANOVA followed by Bonferroni’s multiple comparisons test, ** *p* < 0.01 and *** *p* < 0.001, or unpaired *t* test, * *p < 0.05* and *** *p* < 0.001.

As obesity induces systemic inflammation that can affect the lungs, we first evaluated how obesity affects the lung inflammatory status in naïve mice. We used our previously established diet-induced obesity (DIO) model in C57BL/6J mice [10], based on weight gain following a high-fat diet (HFD) up to 21 weeks of age. Starting at 6 weeks of age, mice were fed either with an HFD (obese) or standard diet (lean), and the bodyweight was documented weekly. Mice fed the HFD rapidly gained weight, and a significant increase over the initial week was observed after 13 weeks of age compared to the mice fed the standard diet (Figure 1A). At the end of the study, the mice on the HFD had ~30% greater body weight than age-matched mice on the standard diet (46.8 g ± 2.101 and 31.4 g ± 0.8902, respectively; Figure 1B), while the lung weight was not altered by diet (0.264 g ± 0.0118 in obese mice, and 0.255 g ± 0.007 in lean mice; Figure 1B).

To study if obesity affected the inflammatory conditions in the lung, inflammatory cells in the bronchoalveolar lavage fluid (BALF) were analyzed. No significant effect of obesity was seen on the BALF total number of inflammatory cells; however, the eosinophil count was significantly reduced in obese mice when compared with lean mice (Figure 1C). The analysis of the immune cells in the lung tissue revealed that obesity did not affect the number of total inflammatory cells, CD4^+^ T cells (Figure 1D), or the Foxp3^+^ Tregs in the lungs (Figure 1E).

### 2.2. AdipoR1-Expressing Tregs Exist in the Lung and Are Reduced in Obese Mice

**Figure 2:**
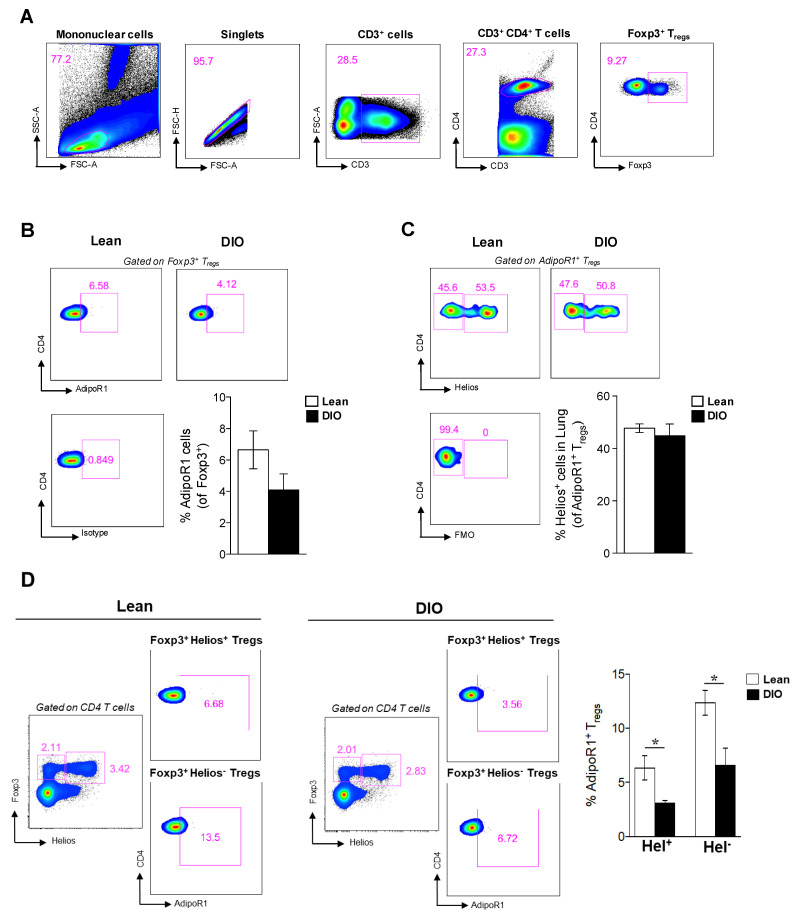
AdipoR1^+^ Foxp3^+^ Tregs were diminished in the lungs of DIO mice at 21 weeks of age. (**A**) The gating strategy of lung Tregs (CD3^+^ CD4^+^ Foxp3^+^). (**B**) Representative plots show the AdipoR1 expression in Foxp3^+^ Tregs and the frequency of AdipoR1-expressing Foxp3^+^ Tregs in the lungs of lean and obese mice. The AdipoR1^+^ population was gated within CD3^+^ CD4^+^ Foxp3^+^ cells. (**C**) Helios expression in lung AdipoR1^+^ Foxp3^+^ Tregs (CD3^+^ CD4^+^ Foxp3^+^ AdipoR1^+^ cells). (**D**) Helios and Foxp3 were used to discriminate subsets of Tregs in lungs of lean and obese mice. Representative plots show the Helios and Foxp3 expression within CD4 T cells and the AdipoR1 expression in Helios^+^ and Helios^−^ Foxp3^+^ Tregs in lean and obese mice. AdipoR1 cells were determined from CD3^+^ CD4^+^ Foxp3^+^ Helios^+^ or CD3^+^ CD4^+^ Foxp3^+^ Helios^−^ cells. Data are means ± SEM of n = 3–6 mice per group. Unpaired *t* test * *p* < 0.05. DIO, diet-induced obesity; AdipoR1, adiponectin receptor 1; and Tregs, regulatory T cells.

Next, we sought to determine whether lung Tregs expressed AdipoR1. The Tregs were determined as CD3^+^ CD4^+^ Foxp3^+^ cells in single cell suspensions of lung tissue (gating strategy in Figure 2A), and the AdipoR1 expression in Foxp3^+^ Tregs was assessed. Lung Tregs expressed AdipoR1 in lean mice, with a reduction in obese mice (6.6% ± 1.204 and 3.7% ± 0.7406, respectively; Figure 2B). To investigate the origin of lung AdipoR1^+^ Tregs, we assessed the expression of Helios. Helios is a transcription factor expressed in a large number of Foxp3^+^ Tregs, and it has been proposed as a marker to distinguish Tregs that developed in the thymus (Helios^+^) and Tregs that were induced from CD4^+^ T cells in peripheral tissues (Helios^−^) [13]. Notably, about half of the lung AdipoR1^+^ Tregs expressed Helios in lean and obese mice (Figure 2C). We next analyzed the expression of AdipoR1 in lung Helios^+^ and Helios^−^ Tregs, which was significantly decreased in obese mice compared to lean mice (Figure 2D). These data indicate that obesity alters the expression of the AdipoR1 receptor in lung Tregs.

### 2.3. Allergic Inflammation Reduces the AdipoR1 Expression in Lung Tregs

**Figure 3:**
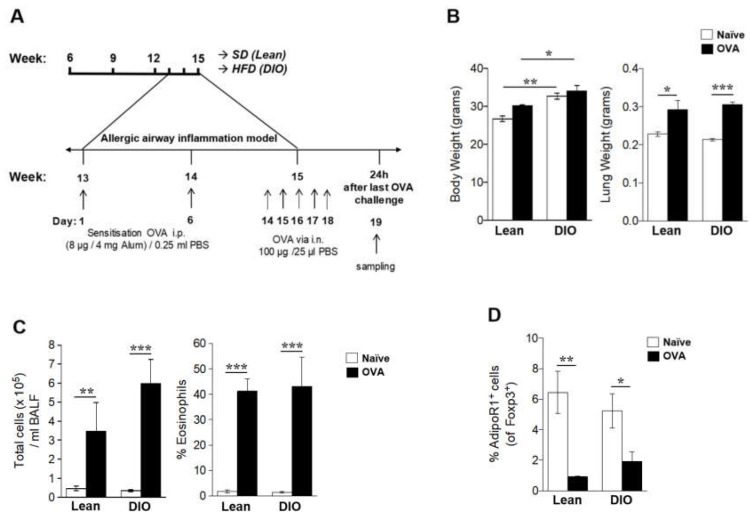
The AdipoR1 expression in lung Foxp3^+^ Tregs was diminished in lungs of DIO naïve and allergic mice. (**A**) The experimental design for the induction of allergic lung inflammation in DIO mice fed with a high-fat diet (HFD) or lean mice fed with a standard diet (SD) up to 15 weeks of age (overweight vs. lean, respectively). Naïve mice were used as controls for the allergic lung inflammation model. (**B**) Terminal body and lung weight at 15 weeks of age. (**C**) Total number of inflammatory cells and eosinophils in bronchoalveolar lavage fluid (BALF). (**D**) Frequency of AdipoR1 in lung Foxp3^+^ Tregs. The AdipoR1^+^ population was gated within CD3^+^ CD4^+^ Foxp3^+^ cells. Data are means ± SEM of n = 3–6 mice per group. Unpaired *t* test * *p* < 0.05, ** *p* < 0.01, and *** *p* < 0.001. DIO, diet-induced obesity; AdipoR1, adiponectin receptor 1; Tregs, regulatory T cells; and OVA, ovalbumin.

To study if allergic inflammation in lean and overweight mice alters the expression of AdipoR1 in lung Tregs, we established a DIO-associated allergic lung inflammation model. Mice on a HFD or SD up to 15 weeks of age (overweight vs. lean mice) were sensitized and challenged with ovalbumin (OVA) (allergic mice), and naïve mice were used as controls (Figure 3A). At the end of the study, both naïve and OVA mice on a HFD exhibited a significant increase in body weight compared with lean mice (Figure 3B). Notably, we found no differences in the lung weight between lean and overweight mice, but a significant increase in the lung weight was observed in OVA mice compared to naïve mice (Figure 3B), reflecting the effect of allergic inflammation. Accordingly, there was a significant increase in the number of BALF inflammatory cells and eosinophils in both lean and obese OVA mice as compared with their respective matched diet naïve mice (Figure 3C). The frequency of AdipoR1^+^ Tregs among regulatory T cells was significantly reduced in both lean and obese OVA mice (Figure 3D).

### 2.4. Obesity Increases Lung Eosinophilia during Allergic Inflammation

**Figure 4:**
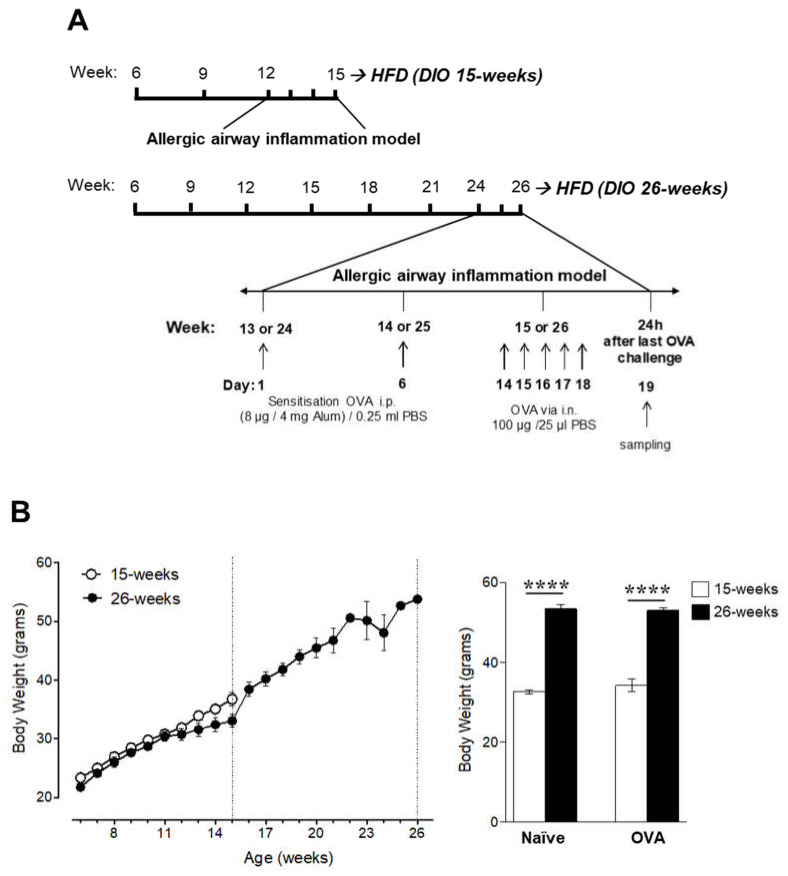

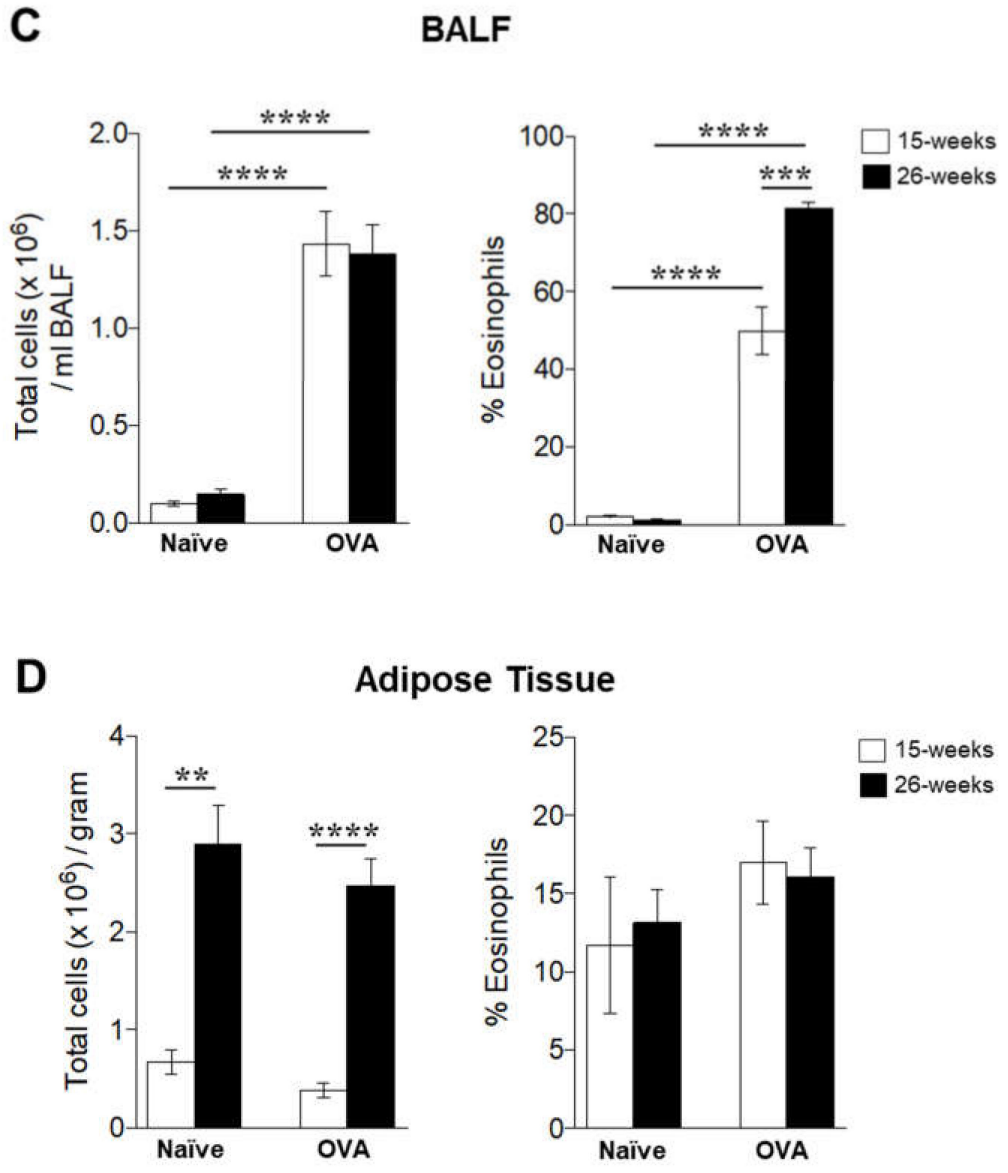
The induction of allergic lung inflammation in DIO mice on different grades of obesity. (**A**) The experimental design for sensitization and challenge with ovalbumin (OVA) in mice on a high-fat diet (HFD) up to 15 weeks of age (overweight model) or 26 weeks of age (morbid obesity model). The model of allergic lung inflammation was induced in DIO mice during the last three weeks of HFD feeding. (**B**) Comparison of the body weight in mice on a HFD up to 15 weeks or 26 weeks of age (left) and the terminal body weight (right). Total inflammatory cells (left) and eosinophils (right) in (**C**) bronchoalveolar lavage fluid (BALF), and (**D**) adipose tissue obtained from DIO naïve and allergic mice. Data are means ± SEM of n = 3–4 mice per group. One-way ANOVA followed by Bonferroni’s multiple comparisons test, ** *p* < 0.01*** *p* < 0.001, and **** *p* < 0.0001. DIO, diet-induced obesity; HFD, high-fat diet; and OVA, ovalbumin.

As increased BMI was linked to increased asthma risk [14], we investigated how additional weight gain affected lung Tregs and their AdipoR1 expression level during allergic airway inflammation. DIO mice with different grades of obesity, i.e., overweight at 15 weeks of age or morbid obesity at 26 weeks of age, were sensitized/challenged with OVA during the last three weeks of HFD feeding (Figure 4A).

Mice fed HFD exhibited a rapid increase in body weight. At the end of the study, mice with morbid obesity at 26 weeks of age had significantly greater body mass than mice of the overweight model at 15 weeks of age, in both naïve and allergic (OVA) mice (Figure 4B). Next, we examined whether the degree of obesity affected the number of inflammatory cells and eosinophils during allergic inflammation. As expected, the number of inflammatory cells and the frequency of eosinophils in the lungs was significantly higher in OVA mice than in the age-matched naïve mice (Figure 4C). The frequency of eosinophils in allergic DIO mice was higher at 26 weeks of age than at 15 weeks of age (82.2% ± 2.250% and 49.8% ± 6.126%, respectively; Figure 4C). As a resident adipose tissue cell, eosinophils have been proposed to redistribute from fat tissues to the lungs [15]. Therefore, we evaluated eosinophils in WAT tissue but neither allergy (OVA) nor the grade of obesity affected the eosinophil frequency in the adipose tissue (Figure 4D). In contrast, the number of inflammatory cells in WAT tissue was significantly higher at 26 weeks of age than at 15 weeks of age (Figure 4D).

### 2.5. Severe Obesity Decreased Lung and Adipose Tissue Helios^+^ Tregs

Several studies have shown that obesity is associated with reduced adipose tissue resident Tregs, which contributes to local and systemic inflammation [9,16]. To investigate whether increased weight gain affected the expression of Helios^+^ and Helios^−^ Foxp3^+^ Tregs in the lungs similarly and how concomitant allergic inflammation modified this, we analyzed cell subpopulations in the lungs of allergic and naïve mice at different grades of obesity as compared to their counterparts in adipose tissue.

We first sought to determine the possible origin of lung and adipose tissue Foxp3^+^ Tregs. Thus, we analyzed the Helios expression in Foxp3^+^ Tregs. Notably, most lung and adipose tissue Foxp3^+^ Tregs obtained from both naïve and allergic DIO mice expressed Helios at 15 and 26 weeks. However, a higher percentage of Helios^+^ cells were observed in the adipose tissue Foxp3^+^ Tregs of DIO mice (Table 1).

**Figure 5:**
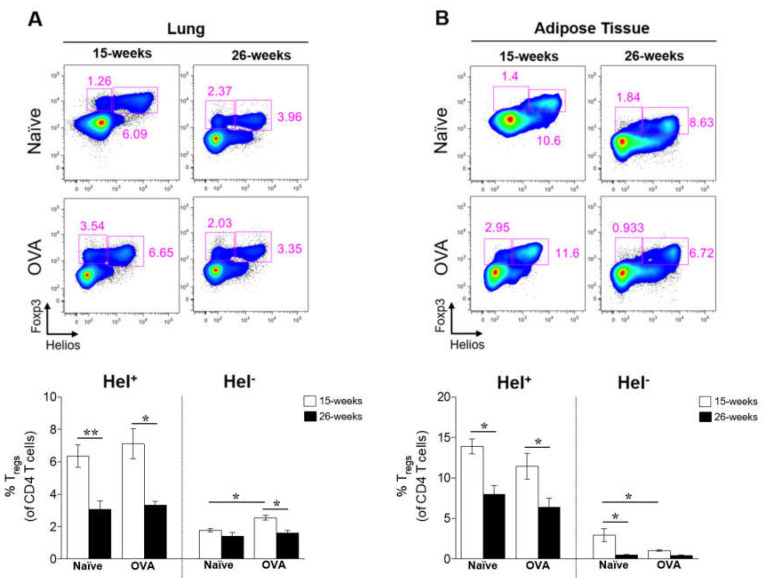
Lung and adipose tissue Helios^+^ and Helios^−^ Foxp3^+^ Tregs in the DIO-associated allergic inflammation model. (**A**) Representative plots show Helios and Foxp3 expression in lung CD4 T cells (upper). The percentages of lung Helios^+^ and Helios^−^ cells within CD4 T cells in naïve and allergic DIO mice on HFD at 15 weeks (overweight) and 26 weeks (morbid obesity) of age (bottom). (**B**) Representative plots show the Helios and Foxp3 expression in adipose tissue CD4 T cells (upper). The percentages of adipose tissue Helios^+^ and Helios^−^ cells within CD4 T cells in naïve and allergic DIO mice (bottom). Subsets of Tregs are showed as CD3^+^ CD4^+^ Foxp3^+^ Helios^+^ or CD3^+^ CD4^+^ Foxp3^+^ Helios^−^ cells. Data are means ± SEM of n = 3–4 mice per group. One-way ANOVA followed by Bonferroni’s multiple comparisons test, * *p* < 0.05, and ** *p* < 0.01. DIO, diet-induced obesity; HFD, high-fat diet; OVA, ovalbumin; AdipoR1, adiponectin receptor 1; Tregs, regulatory T cells; and Hel, Helios.

Evaluating the effects of weight gain and concomitant allergic inflammation, flow cytometry analysis revealed a significant reduction in Helios^+^ Foxp3^+^ Tregs in the lungs of both allergic and naïve obese mice at 26 weeks of age compared to allergic and naïve obese mice at 15 weeks of age (Figure 5A). Similarly, adipose tissue Helios^+^ Foxp3^+^ Tregs were significantly decreased in response to obesity (Figure 5B).

No differences were found in the Helios^−^ Foxp3^+^ Tregs in the lungs of naïve mice between 15 and 26 weeks, whereas the presence of allergic inflammation resulted in a small but significant reduction (Figure 5A). In contrast, upon weight gain, Helios^−^ Foxp3^+^ Tregs were reduced in the adipose tissue of naïve mice, but not of mice with allergic inflammation (Figure 5B).

### 2.6. Lung and Adipose Tissue adipoR1^+^ Tregs Are Differentially Regulated by Obesity

**Figure 6:**
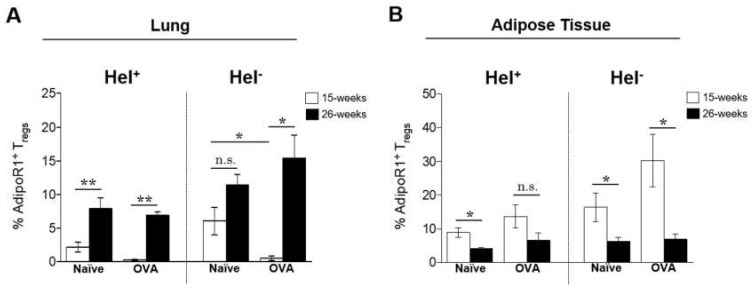
Lung and adipose tissue AdipoR1^+^ Tregs were differentially regulated in the DIO-associated allergic inflammation model. (**A**) AdipoR1 expression in lung Helios^+^ and Helios^−^ Foxp3^+^ Tregs in naïve and allergic DIO mice on HFD at 15 weeks (overweight) and 26 weeks (morbid obesity) of age. (**B**) AdipoR1 expression in adipose tissue Helios^+^ and Helios^−^ Foxp3^+^ Tregs in DIO naïve and allergic mice. AdipoR1^+^ population gated within CD3^+^ CD4^+^ Foxp3^+^ Helios^+^ or CD3^+^ CD4^+^ Foxp3^+^ Helios^−^ cells. Data are means ± SEM of *n* = 3–4 mice per group. One-way ANOVA followed by Bonferroni’s multiple comparisons test, *p <0.05, and **p < 0.01. DIO, diet-induced obesity; HFD, high-fat diet; OVA, ovalbumin; AdipoR1, adiponectin receptor 1; Tregs, regulatory T cells; and Hel, Helios.

Next, we examined the effect of excessive weight gain on the expression of AdipoR1 in Foxp3^+^ Tregs in the lung and the adipose tissue. Weight gain resulted in a marked increase in AdipoR1 expression in both Helios^+^ and Helios^−^ lung Tregs in both naïve and allergic mice (Figure 6A). The AdipoR1 expression in Helios^−^ Tregs was significantly reduced in allergic mice at 15 weeks of age compared to their matched-age naïve controls (Figure 6A).

In adipose tissue, the contrary, extensive weight gain resulted in decreased expression of AdipoR1 in both Helios^+^ and Helios^−^ Tregs (Figure 6B), whereas the allergic inflammation in morbidly obese mice (at 26 weeks) did not alter in the expression of AdipoR1 in either lung or adipose tissue Helios^+^ and Helios^−^ Tregs, as no differences were found between naïve and OVA mice (Figure 6B).

### 2.7. AdipoR1 Expression in Lung Tregs Is Associated with Body Weight

We showed previously that the AdipoR1 expression in adipose Tregs was associated with the epididymal fat volume [10]. Thus, we next evaluated whether the AdipoR1 expression in lung Tregs was associated with the grade of obesity.

**Figure 7:**
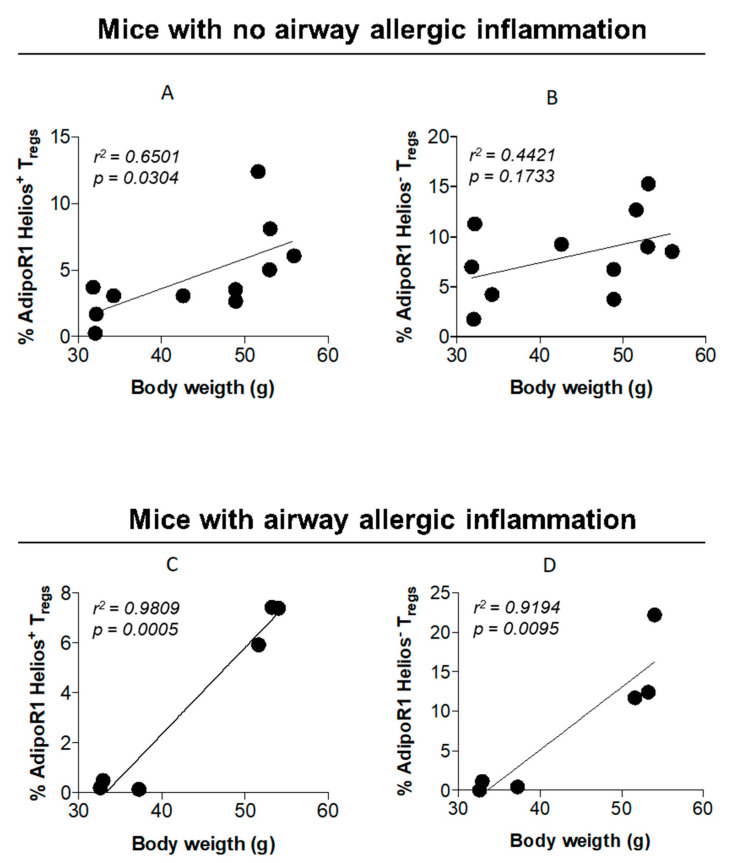
The AdipoR1 expression in lung Tregs was correlated with the grade of obesity during allergic inflammation. Scatter graphs showing the correlation analysis between the AdipoR1 expression in Helios^+^ (**A**) and Helios^−^ (**B**) Tregs and the body weight in naïve (non-allergic lung inflammation) mice on HFD at 15, 21, and 26 weeks of age, and Helios^+^ (**C**) and Helios^−^ (**D**) in allergen-induced airway inflammation model in mice on HFD at 15 and 26 weeks of age. Data are means ± SEM of n = 6–11 mice per group. *r^2^*, Pearson correlation coefficient; HFD, high-fat diet; AdipoR1, adiponectin receptor 1; Tregs, regulatory T cells; and Hel, Helios.

Mice at different stages of weight gain (15, 21, and 26 weeks; overweight, obese, and morbidly obese models, respectively) with no allergic inflammation and mice with different stages of weight gain (15 and 26 weeks; overweight and morbidly obese models, respectively) were investigated regarding airway allergic inflammation. In non-allergic mice, the AdipoR1 expression was correlated with the body weight in lung Helios^+^ Tregs (Figure 7A). In contrast, in mice with allergic lung inflammation, a marked correlation was found in both Helios^+^ and Helios^−^ lung Tregs (Figure 7B).

## 3. Discussion

The present study is the first to describe that lung regulatory T cells express the AdipoR1 receptor and describe how different levels of weight gain and airway allergic inflammation affect this expression. Using models of DIO and airway allergic inflammation, we showed that obesity resulted in a reduction in the AdipoR1 expression. However, when the weight gain was excessive, as in morbid obesity, we found an increased expression. Airway allergic inflammation in lean mice also resulted in a reduced expression, while for airway allergic inflammation combined with obesity, the AdipoR1 expression followed the obesity pattern.

Further significant findings include the effects of obesity and airway allergic inflammation in lung eosinophilia and Helios^+^ and Helios^−^ regulatory T cells. While obesity resulted in reduced airway eosinophilia in naïve mice, allergic inflammation had similar effects in overweight and lean mice. We showed that excessive weight gain, such as in morbid obesity, resulted in a significant increase in allergy-induced airway eosinophilia. Finally, we showed for the first time that, in this very same case of morbid obesity, lung, and adipose tissue Helios^+^ and Helios^−^ regulatory T cells were affected similarly following weight gain, increased age, and airway allergic inflammation.

In lean adipose tissue, the eosinophils and Tregs, the prominent resident T cell population, are responsible for polarizing adipose tissue macrophages to an anti-inflammatory phenotype through the secretion of anti-inflammatory cytokines. Adiponectin is the primary anti-inflammatory adipokine secreted by adipocytes that exerts its actions by binding to the adiponectin receptors AdipoR1 and AdipoR2 [17]. During obesity, circulating levels of adiponectin are reduced, while low adiponectin levels have been associated with asthma in adults [11,18]. All the above proposed for a role for Tregs and adiponectin in obese-related asthma. Our results confirm this hypothesis. We identified that Tregs expressed AdipoR1 in lean mice and that half of them expressed the transcription factor Helios, a proposed marker for the thymus derived Tregs [13].

We showed that weight gain in an overweight model (15 weeks) resulted in decreased AdipoR1 expression in Tregs. A similar effect was also found during the airway allergic inflammation. The role of AdipoR1 expression in Tregs is still unclear but previous studies have shown that adiponectin regulates Th17 differentiation [19,20]. Those data argue for a role of AdipoR1 in modulating the Treg response, particularly as Th17 and Tregs have a common progenitor and show a high grade of plasticity during inflammatory conditions [21]. The role of IL-17 in asthma has gained increased interest lately, where even a protective role is proposed [22]. When extensive weight gain occurs, as in the model of morbid obesity (26 weeks), we found an increase in AdipoR1. Correlation between AdipoR1 expression and weight gain was stronger in mice with allergic inflammation, proposing an augmented role of adiponectin in allergic inflammation. Indeed, adiponectin is a pleiotropic adipokine, and its circulating levels are decreased mainly at morbid obesity [23] and play a role in allergic inflammation [24]. Thus, a possible explanation for this diverse expression might be that the AdipoR1 expression is upregulated in response to decreased circulating adiponectin levels.

A critical difference between the above two mouse models is the age, from 15 to 26 weeks. Tregs are described to be regulated in an age-dependent manner. Both thymus-derived Tregs (i.e., Helios expressing) as well peripheral-induced Tregs (i.e., Helios negative) decrease with age [25]. Our data align with the above observations as mainly Helios^+^ Tregs were decreased in the lungs between 15 and 26 weeks. Thus, the decrease we see from 15 to 26 weeks can be due to both extensive obesity and age. Our current data do not allow us to exclude the age effect. However, we observed a parallel pattern in the adipose tissue. Extensive weight gain resulted in decreased Tregs in adipose tissue, a phenomenon well described earlier [26], thus, allowing us to focus on the extensive weight gain.

Airway eosinophilia represents a hallmark of airway allergic inflammation [27]. However, there is a debate if, in obese individuals with asthma, allergic inflammation is dominated by eosinophils or neutrophils. Obesity-related asthma has been correlated earlier with a dominance of a neutrophilic airway inflammation [28]. However, studies have shown the opposite, in that obese individuals with severe [29] and mild-to-moderate asthma [30] demonstrated increased eosinophils in lung biopsies. In our models, we found that obesity resulted in a decreased airway eosinophilia in naïve mice. Interestingly in our overweight model, both lean and overweight mice had similar airway eosinophil levels.

Airway allergic inflammation induced a significantly enhanced airway eosinophilia in mice within the morbid-obesity model compared to the overweight model mice. However, as both the sensitization and challenge were performed in the last 14 days of the high-fat diet, the effect of obesity on the sensitization and challenge cannot be separated. Future studies will be performed to answer this important question and further queries related to the Th2 response. All the above data suggest that although obesity per se reduced airway eosinophilia, extensive obesity promotes allergic airway eosinophilia, supporting the earlier studies in humans. Those findings are also in line with the previous findings where obesity was found to be a risk factor for developing asthma and other allergic diseases, including allergic rhinitis and atopic dermatitis [31,32].

As eosinophils play an anti-inflammatory role in lean and “healthy” adipose tissue, it has been proposed that eosinophils migrate from adipose tissue to the airways in obese individuals with asthma [33]. To evaluate this in our morbid-obesity model, we also evaluated adipose tissue eosinophils and found no effect either by weight gain or allergic inflammation, thus, indicating that airway eosinophilia originated outside the adipose tissue. Our findings show an increase in eosinophils in allergic airways together with a decrease in the fat tissue, supporting findings from an earlier study reporting that diet-induced obesity enhanced eosinophil trafficking from the bone marrow to the lung tissue [34].

Our study has limitations. This study was mainly a descriptive study with a lack of functional data. Unanswered questions include how the levels of type 2 inflammation, such as cytokines IL4, IL13, IL5, and adiponectin levels, both systemically but also locally in the airways, are affected in our model from the different stages of obesity and allergic inflammation. At the same time, the results will need to be validated in human samples. Both goals are beyond the scope of this work, in which the main objective was to evaluate the presence of AdipoR1 expression in lung Tregs.

In conclusion, we demonstrated that Tregs in the lungs expressed AdipoR1, which was modulated by obesity and allergic inflammation, and that airway eosinophilia increased with excessive weight gain. These main findings link two central players in inflammatory responses, Tregs and adiponectin, to airway eosinophilia and obesity-associated asthma. An important future direction includes the validation of our findings in human samples with an overall aim to evaluate the therapeutic potential of adiponectin in asthma.

## 4. Materials and Methods

### 4.1. Mice

Male C57BL/6J mice at 5 weeks of age were obtained from Charles River (Sulzfeld, Germany) and maintained under standard housing conditions with food and water ad libitum. All animal protocols were approved by the Animal Ethics Committee in Gothenburg, Sweden (permit number Dnr 210-2012).

### 4.2. Diet-Induced Obesity (DIO) Models

Mice at 6 weeks of age were fed with a high-fat diet (HFD) (D12492; Research Diet, New Brunswick, NJ, USA; carbohydrate: 20%; protein: 20%; fat: 60%) to induce obesity. Animals were fed up to 15 weeks of age (overweight model), 21 weeks of age (obesity model), and 26 weeks of age (morbid obesity model). Age-matched mice were fed on standard-chow diet and used as lean controls. The body weight was recorded once a week throughout the study.

### 4.3. Induction of Allergic Airway Inflammation in DIO Mice

Allergic airway inflammation was induced in overweight and morbid obesity mouse models at 13 or 24 weeks of age. The animals were sensitized twice (Day 1 and Day 6) by intraperitoneal injections of 8 μg of ovalbumin (OVA; Sigma-Aldrich, St Louis, MO, USA) bound to 4 mg of aluminum hydroxide (Sigma-Aldrich) in 0.25 mL of sterile PBS. Acute allergic inflammation was induced by five consecutive intranasal challenges of OVA (Day 14 to Day 18). At each challenge, obese animals were briefly anesthetized using isoflurane (Flurane^®^, Baxter, Deerfield, IL, USA), and 100 μg of OVA was administrated in 25 μL of sterile PBS. During the induction of allergic inflammation, DIO mice were maintained on a HFD up to 15 weeks (overweight model) and 26 weeks (morbid obesity model) of age. Age-matched DIO non-sensitized mice were used as naïve controls [35].

### 4.4. Tissue Collection and Preparation of Single-Cell Suspensions

Lung and adipose tissue were collected 24 h after the last OVA challenge. The mice were euthanized with a mixture of xylazin (130 mg/kg; Rompun, Bayer, Leverkusen, Germany) and ketamine (670 mg/kg; Ketalar, Apoteket AB, Motala, Sweden). Lungs without any connective tissue and epididymal white adipose tissue (WAT) were removed and placed in Hanks balanced salt solution (HBSS^+^; Gibco, Paisley, UK). The tissues were weighed and kept on ice until the preparation of lung single-cell suspensions. The lung tissue was dissociated using a gentleMACS Dissociator (Milteny Biotec GmbH, Bergisch Gladbach, Germany) according to the manufacturer’s instructions. In brief, the lungs were transferred into a gentleMACS C Tube containing Dulbecco’s modified Eagle’s medium high glucose (HyClone, Logan, UT, USA), 10% heat-inactivated FBS (Sigma-Aldrich), 1% penicillin/streptomycin (HyClone), 50 mM 2-mercaptoethanol (Sigma-Aldrich), 25 U/mL DNase I (Roche, Applied Science, Mannheim, Germany), and 250 U/mL collagenase type IV (Life Technologies, Gibco, Grand Island, NY, USA) and digested for 20 min at 37 °C. The adipose tissue was cut into small pieces, digested in RPMI 1640 (HyClone), 5% heat-inactivated FBS (Sigma-Aldrich), 1% penicillin/streptomycin (HyClone), 0.1% DNase I (Roche), and 1.6 mg/mL collagenase type IV (Gibco), and digested for 1 h at 37 °C. The resulting cell suspension was then filtered, and the stromal vascular fraction was collected. Single cell suspensions were washed and counted using a hemocytometer before being stained for FACS analysis.

### 4.5. Cytospins

A total of approximately 50,000 lung and adipose tissue single-cell suspensions in 100 μL PBS were used for cytospins. The cells were stained with May-Grünwald-Giemsa (HistoLab Products AB, Gothenburg, Sweden) according to the manufacturer’s protocol. The samples were evaluated using a light microscope (Zeiss Axioplan 2, Carl Zeiss, Germany) at a magnification of 100×. Differential cell counting was performed based on the morphologic criteria, and 300 cells were counted per slide.

### 4.6. Flow Cytometry

Single-cell suspensions were suspended in FACS buffer and pre-incubated with 2% mouse serum (Dako, Glostrup, Denmark) for 15 min. Then, the cells were stained with anti-CD3e (Clone 145-2C11, BD Biosciences, San Jose, CA, USA), anti-CD4 (Clone GK1.5, BD Biosciences), and anti-AdipoR1 (Phoenix Pharmaceuticals, Burlingame, CA, USA) antibodies. For AdipoR1 detection, a secondary antibody was added (Clone Poly 4064, BioLegend, San Diego, CA, USA) and incubated for an additional 20 min. Intracellular staining was performed using a Foxp3 staining buffer set (eBioscience, San Diego, CA, USA) according to the manufacturer’s instructions. To detect intracellular markers, the cells were stained with anti-Foxp3 (Clone 236A/E7, eBioscience), and anti-Helios (Clone 22F6; eBioscience) antibodies. The cells were processed on a BD FACSVerse Flow Cytometer running BD FACSuite Software (BD Biosciences) and the data were analyzed with FlowJo 9.3.2 software (TreeStar, Inc., Ashland, OR, USA). T helper cells were identified as CD3^+^ CD4^+^ cells from a mononuclear cell gate. Treg cells were identified as CD3^+^ CD4^+^ Foxp3^+^ that either co-expressed Helios or did not. The gating of markers was determined using the Fluorescence Minus One (FMO) approach. AdipoR1 positive cells were determined using the FMO approach but with adding the secondary antibody. The gating of cell populations is shown in the figure legends.

### 4.7. Statistical Analysis

The results are expressed as mean ± SEM. Statistical analysis was performed with GraphPad Prism version 5.0 (GraphPad Software, La Jolla, CA, USA). One-way or two-way ANOVA followed by Bonferroni’s multiple comparisons test, or an unpaired *t* test was performed. The association between the AdipoR1 expression in Treg subsets and the body weight was evaluated using Pearson’s correlation analysis. The significance for all statistical tests is shown in the figures as * *p* < 0.05, ** *p* < 0.01, *** *p* < 0.001, and **** *p* < 0.0001.

## Figures and Tables

**Table 1:** The expression of Helios in lung and adipose tissue Foxp3^+^ Tregs from the DIO mouse model at 15 and 26 weeks.

		Lung	Adipose	Tissue
	Overweight model	Morbid obesity model	Overweight model	Morbid obesity model
	15 weeks	26 weeks	15 weeks	26 weeks
	(% of Foxp3^+^ cells)	(% of Foxp3^+^ cells)	(% of Foxp3^+^ cells)	(% of Foxp3^+^ cells)
Naive	66.87 ± 0.15	65.89 ± 1.187	84.17 ± 3.209	91.98 ± 0.981
OVA	72.96 ± 1.831	65.07 ± 0.484	83.18 ± 2.498	92.25 ± 1.418

DIO, diet-induced obesity; OVA, ovalbumin; AdipoR1, adiponectin receptor 1; and Tregs, regulatory T cells.

## Abbreviations

AdipoR1	Adiponectin receptor 1
BALF	Bronchoalveolar lavage fluid
BMI	Body mass index
DIO	Diet-induced obesity
Foxp3	Forkhead box P3
Hel	Helios
HFD	High-fat diet
OVA	Ovalbumin
PBS	Phosphate buffer saline
WAT	White adipose tissue
